# Supplementary material for: Splice-Junction-Based Mapping of Alternative Isoforms in the Human Proteome
Source: Cell Rep. Author manuscript; Available in PMC 2020 Jan 15. (PMC6961840; doi:10.1016/j.celrep.2019.11.026)
Supplement: 2 [file NIHMS1546469-supplement-2.zip › DF1/PXD006675/RightCoronaryArtery/RightCoronaryArtery_16_PRDX5_AAAMAPIKTHLPGFVEQAEALK.pdf]

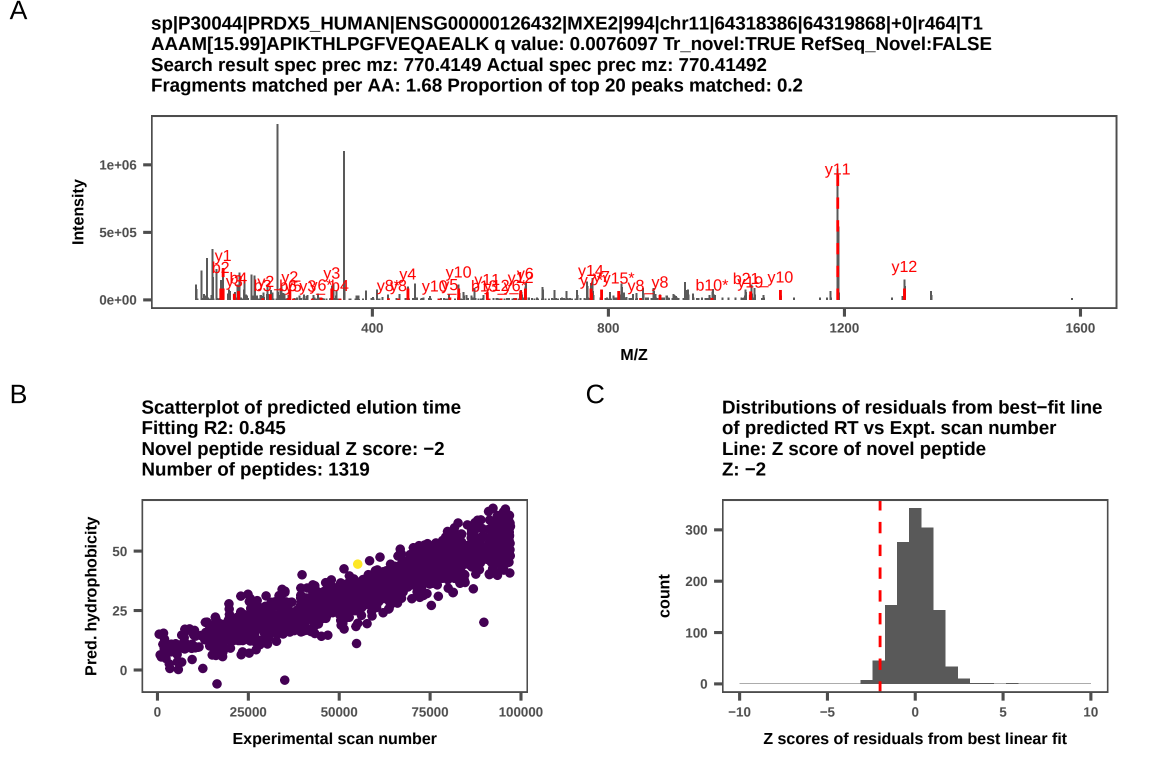
A
sp|P30044|PRDX5_HUMAN|ENSG00000126432|MXE2|994|chr11|64318386|64319868|+0|r464|T1
AAAM[15.99]APIKTHLPGFVEQAEALK q value: 0.0076097 Tr_novel:TRUE RefSeq_Novel:FALSE
Search result spec prec mz: 770.4149 Actual spec prec mz: 770.41492
Fragments matched per AA: 1.68 Proportion of top 20 peaks matched: 0.2
Intensity
1e+06
5e+05
0e+00
y11
y12
y10
b10*
y1
y3
y4
y8
M/Z
400
800
1200
1600
B
Scatterplot of predicted elution time
Fitting R2: 0.845
Novel peptide residual Z score: −2
Number of peptides: 1319
Pred. hydrophobicity
50
25
0
0
25000
50000
75000
100000
Experimental scan number
C
Distributions of residuals from best−fit line
of predicted RT vs Expt. scan number
Line: Z score of novel peptide
Z: −2
count
300
200
100
0
−10
−5
0
5
10
Z scores of residuals from best linear fit
